# Senescence and Host–Pathogen Interactions

**DOI:** 10.3390/cells9071747

**Published:** 2020-07-21

**Authors:** Daniel Humphreys, Mohamed ElGhazaly, Teresa Frisan

**Affiliations:** 1 Department of Biomedical Science, University of Sheffield, Sheffield S10 2TN, UK; melghazaly1@sheffield.ac.uk; 2 Department of Molecular Biology, Umeå University, 901 87 Umeå, Sweden; 3 Umeå Centre for Microbial Research (UCMR), Umeå University, 901 87 Umeå, Sweden

**Keywords:** viral and bacterial infections, DNA damage, senescence, microbial replication, bacterial genotoxins, cancer, tissue microenvironment

## Abstract

Damage to our genomes triggers cellular senescence characterised by stable cell cycle arrest and a pro-inflammatory secretome that prevents the unrestricted growth of cells with pathological potential. In this way, senescence can be considered a powerful innate defence against cancer and viral infection. However, damage accumulated during ageing increases the number of senescent cells and this contributes to the chronic inflammation and deregulation of the immune function, which increases susceptibility to infectious disease in ageing organisms. Bacterial and viral pathogens are masters of exploiting weak points to establish infection and cause devastating diseases. This review considers the emerging importance of senescence in the host–pathogen interaction: we discuss the pathogen exploitation of ageing cells and senescence as a novel hijack target of bacterial pathogens that deploys senescence-inducing toxins to promote infection. The persistent induction of senescence by pathogens, mediated directly through virulence determinants or indirectly through inflammation and chronic infection, also contributes to age-related pathologies such as cancer. This review highlights the dichotomous role of senescence in infection: an innate defence that is exploited by pathogens to cause disease.

## 1. Introduction

Cellular senescence is a process by which cells undergo stable cell cycle arrest and phenotypic changes, including modifications of chromatin and a pro-inflammatory secretome that prevents the unrestricted growth of damaged cells [1,2]. Consequently, senescence represents an important innate defence against pathologies that hijack cellular proliferation to cause disease. Different types of senescence have been identified such as replicative senescence (RS), oncogene-induced senescence (OIS), genotoxicity-induced senescence (GIS), developmental senescence (occurring during embryogenesis) and tissue repair senescence [2,3,4].

OIS represents a major tumour suppressor mechanism while senescence can also restrict viral replication. Senescent cells release a pro-inflammatory secretome that underlies the senescence-associated secretory phenotype (SASP), which impacts diverse biological processes in the tissue microenvironment through paracrine signalling [5]. SASP can induce paracrine senescence in bystander cells, as well as the inflammation and infiltration of innate immune cells that further restrict the threat posed by cells with pathological potential [5,6,7].

The rapid ‘acute’ forms of senescence, in response to overwhelming DNA damage, represent a safeguard against the ill health of an organism [8]. Other beneficial roles of acute senescence include development, tissue homeostasis and wound healing where myofibroblasts undergo acute senescence to limit scarring [8,9]. However, there are two sides to cellular senescence, which can also cause disease. This is exemplified by acute OIS [7,10] and chronic senescence caused by cells experiencing mild and persistent genotoxic stress [8,11]. Chronic senescence is detrimental to an organism and contributes to age-related pathologies such as cancer, cardiovascular disease, diabetes, and neurodegenerative disorders [8,12,13,14]. Ageing cells accumulate macromolecular damage, which leads to the stochastic build-up of senescent cells and chronic inflammation via SASP resulting in genomic instability and pathology [12,14,15]. Thus, senescence can be viewed as a good servant but a bad master that can tip the balance in favour of disease.

Pathogens are masters of subverting biological processes for their own gain during infection, yet it was not until relatively recently that senescence has emerged as a possible hijack target [16,17,18,19,20,21,22,23]. Bacterial and viral pathogens possess armouries of virulence factors that have the potential to induce senescence responses either directly (e.g., dedicated bacterial toxins) or indirectly (e.g., fundamental pathogen-associated molecular patterns such as viral capsids, bacterial flagella and lipopolysaccharide) [23]. In this review, we discuss senescence as an emerging hijack target of pathogens and consider how collateral damage resulting from infection might contribute to age-related diseases. We discuss senescence in the context of combatting pathogens and mobilising innate immune responses, and incorporate the lessons learnt from viral infections to present a current view on the senescence host–pathogen interaction.

## 2. Hallmarks of Cellular Senescence

The cellular and molecular features of senescent cells are complex and can vary depending on several parameters such as the cell type, stressor, intensity and length of the stimulus.

Four key parameters have been identified as associated with the senescent phenotype: irreversible cell cycle arrest, the secretion of soluble mediators and extracellular vesicles (SASP or senescence messaging secretome, SME), the damage of macromolecules (DNA, proteins and lipids) and altered metabolism ([2]). Considering the multiple triggering factors, the characterisation of senescent cells is not unequivocal and requires a multi-marker approach, as summarised in ([2]).

**Figure 1:**
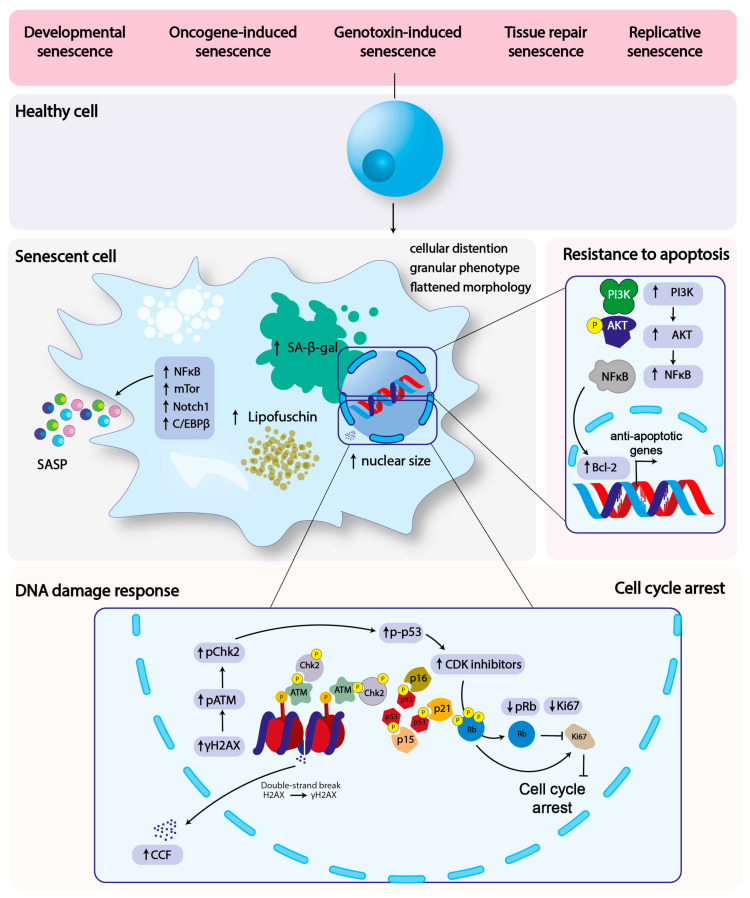
**Senescence-triggering factors and the features of senescent cells**. The figures summarised the four key features of senescence relevant for the host–pathogen interaction (cell cycle arrest, DNA damage response, SASP and resistance to apoptosis), and the key molecules involved in each of these processes. Senescence can be induced by several stimuli, such as telomeres erosion (replicative senescence), the over-activation of oncogenes, which will induce replication stress and consequent chronic DNA damage (oncogene-induced senescence), chronic DNA damaged caused by exogenous or endogenous genotoxic agents (e.g., chemotherapy, irradiation, oxidative stress, genotoxic-induced senescence). Physiological types of senescence also occur during embryonic development (developmental senescence) or tissue repair.

In this section, we will focus on the aspects of senescence relevant for host–pathogen interaction: cell cycle arrest, DNA damage, resistance to apoptosis and SASP (Figure 1).

The key feature of senescent cells is their permanent exit from the cell cycle progression, the enhanced activity of the lysosomal senescence-associated β-galactosidase (SA-β-gal), the acquisition of a flatten morphology, granular phenotype, and enlarged nucleus [2,24].

The permanent cell cycle arrest in senescent cells is dependent on the activation of the cyclin kinase inhibitors p21^WAF/Cip1^ (CDKN1A), p16^INKA^ (CDKN2A) and p15^INK4b^ (CDKN2B).

The p21 is a transcriptional target of p53 and a potent inhibitor of several cyclin/cyclin kinase complexes, including cyclins E/A-CDK2, cyclin A-CDK1, and cyclin Ds-CDK4,6, therefore p21 promotes cell cycle arrest in different phases of the cell cycle, depending of the cyclin/CDK complex targeted. p16 and p15 inhibit the cyclin Ds-CDK4,6 resulting in the accumulation of the hypophosphorylated form of the tumour suppressor retinoblastoma (RB) protein, arresting the cell at the G1/S transition [25,26,27].

The presence of persistent DNA damage is a strong trigger of senescence. RS is caused by telomere shortening during cellular replication, which results in the telomere uncapping and activation of the DNA damage response (DDR) [28]. Conversely, OIS is the consequence of the sustained replication promoted by oncogene overexpression, which leads to the collapse of the replication forks, the induction of DNA damage and DDR activation, a phenomenon considered a powerful barrier for cancer development [29]. In addition, chronic exposure to endogenous or exogeneous DNA damaging agents, such as ionizing and ultraviolet radiations, oxidative stress, chemotherapeutics, and bacterial toxins, triggers senescence [30]. The chronic activation of DDR can be visualised at the DNA damaged site by the detection of the phosphorylated form of the ataxia telangectasia mutated (ATM) kinase (Ser1981), histone H2A phosphorylation (γH2AX, Ser 139), p53 (Ser15), CHK2 (Thr68), the recruitment of adaptor and repair proteins such as MDC1, NBS1, MRE11, 53BP1, CHK2, and at a later stage of the promyelocytic leukaemia protein (PML) nuclear bodies, in structures known as DNA fragments with chromatin alteration reinforcing senescence (DNA-SCARS) [31].

Another type of DNA damage detected in senescent cells is the presence of cytoplasmic chromatin fragments (CCFs). Due to the unusual location, CCFs are perceived by the cell as a threat, triggering the activation of the cGAS-STING-mediated innate response, leading to the production and secretion of type I interferon (IFN-I) [32,33,34,35], thus contributing to the secretory phenotype of senescent cells (see below).

The permanent quiescent state of senescent cells contrasts with their capacity to secrete a plethora of mediators (namely SASP) contributing to the remodelling of the surrounding microenvironment in a paracrine and juxtacrine manner [7,36,37,38,39].

SASP is regulated by the activation of transcription factors and signalling pathways, many of these associated with the induction and secretion of pro-inflammatory mediators (e.g., IL-1α, IL-6, IL-8, growth factors, and extracellular matrix metalloproteases), including NFκB and C/EBPβ, the inflammasome, the p38 MAPK and mTOR pathways [7,40,41]. However, recent evidence indicates that the composition of SASP can differ, depending on the genetic background or the time after the senescence is triggered. Indeed, the JAK2-activated STAT3 transcription factor promotes an immune suppressive SASP in phosphatase and angiotensin homologue (PTEN)-deficient prostate cancer cells, characterised by secretion of IL-10 and IL-13 [42]. On the other hand, oncogene- or DNA damage-induced senescence induces the transient activation of the NOTCH1 signalling pathway in human diploid fibroblasts, leading to the suppression of pro-inflammatory cytokine production, via the inhibition of C/EBPβ [39]. These data indicate that SASP composition and the consequent modulation of the microenvironment is complex, possibly due to different SASPs, depending on the temporal and spatial induction of senescence. This feature may be exploited by microorganisms to manipulate the host microenvironment in order to establish a successful infection.

Several lines of evidence demonstrate that senescent cells are more resistant to apoptosis in response to genotoxic, oxidative and endoplasmic reticulum (ER) stress [24]. This resistance is dependent on the activation of several pathways, and considering the heterogenicity of the triggering signals, the survival mechanisms may be different in the different types of senescence. DNA damage leads to the activation of the transcription factor NFκB, which is a key contributor to the SASP but also upregulates the expression of pro-survival genes such as BCL-2 [43]. DNA damage as well as growth factors (e.g., IGF-1) secreted by the senescent cells activate the PI3K–AKT axis, which results in (i) the phosphorylation of pro-apoptotic protein BAD and its dissociation from the BAD/Bcl-X_L_ complex [44]; (ii) the phosphorylation and inactivation of the transcription factor FOXO3, and the consequent downregulation of the pro-apoptotic BH3-only members of the BCL2 family (BIM BIK, NOXA, PUMA) [45].

The secretory phenotype of senescent cells is associated with intense ER stress, due to the accumulation of misfolded proteins triggering a process known as unfolded protein response (UPR) that results in the remodelling and expansion of the ER membrane to cope with the high protein secretion rate [24,46].

## 3. Infection and Senescence

**Figure 2:**
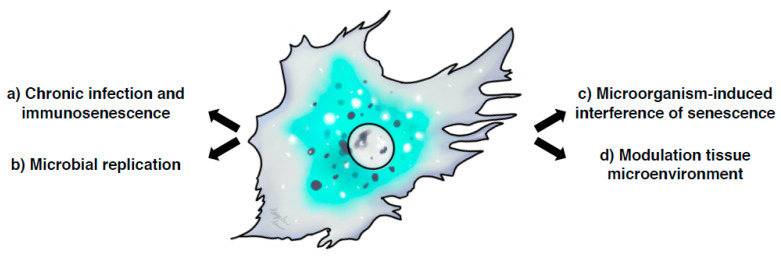
**Crosstalk between cellular senescence and infections.** Several aspects of senescence can be highlighted in infections: (**a**) immunosenescence due to chronic infections, which can impair the host immune responses and contribute to persistent infections; (**b**) the regulation of microbial replication and invasion where senescent cells can either enhance microbial replication or act as innate immune defence mechanisms limiting the rate of infection; (**c**) the microorganism-induced senescence, due to the direct induction of chronic DNA damage either indirectly via chronic inflammation and consequent oxidative stress, or directly by the production of genotoxins; (**d**) the senescence and modulation of the tissue microenvironment, which can contribute to the establishment of a favourable niche to ensure a successful infection.

Several aspects of senescence can be highlighted in infections: (a) immunosenescence due to chronic infections; (b) regulation of microbial replication and invasion; (c) microorganism-induced interference of senescence; (d) senescence and modulation of the tissue microenvironment (Figure 2). In the next section, we will discuss these issues in the context of viral and bacterial infections.

### 3.1. Immunosenescence

Aging is associated with a progressive loss of the immune system efficacy, which is detected both within the innate immune compartment, and most prominently, in the effector cells of the adaptive immune response: B and T lymphocytes [47,48].

Professional phagocytic cells, such as neutrophils and macrophages, show decreased phagocytic capacity and oxidative burst. Parallel to the decline of these key antimicrobial effector functions, peripheral blood mononuclear cells from older individuals secrete higher amounts of pro-inflammatory cytokines, including IL-1, IL-12 and TNF-α compared to the levels observed in the cells isolated form younger donors [49,50], a condition that has been epitomised by the term ‘inflammageing’ [51,52].

Hallmarks of immunosenescence in the T and B cell compartment are: (i) the reduced ability to respond to new antigenic stimuli; (ii) the accumulation of memory T cells (a process known as memory inflation); (iii) the reduced diversity of the T and B cell receptors; (iv) the impaired ability of B cells to produce high affinity antibodies; (v) the reduced serum levels of antibodies produced by naïve B lymphocytes (IgD and IgM) [47,48].

Immunosenescence has been associated with several chronic viral infection, including human cytomegalovirus (HCMV), human hepatitis C virus (HCV), human immunodeficiency virus (HIV) and human acute T cell leukaemia virus type I (HTLV-1).

#### Immunosenescence and Chronic Viral Infections

HCMV is an enveloped double stranded DNA beta herpes virus that establishes a latent infection, with periodic reactivation controlled by the host cytotoxic T lymphocytes (CTL) response in healthy individuals [53]. Continued antigen exposure as seen in the HCMV infection (hyperantigenemia) results in telomere erosion and replicative senescence, a process which occurs faster and more frequently in CD8+ cytotoxic T lymphocytes (CTL) compared to CD4+ T helper cells [54,55,56].

HCMV-specific CD8+ and CD4+ T lymphocytes exhibit features of immunosenescence characterised by CD57 expression and reduced levels of CD27 and CD28. However, there is not a general consensus whether this phenotype is associated with reduced telomere length [57,58,59]. HCMV-specific T lymphocytes can secrete higher levels of pro-inflammatory cytokines (IFN-γ and TNF-α) upon stimulation [56], possibly contributing to the process of inflammageing, but exhibit poor proliferative responses, possibly due to the eroded feature of their telomeres.

It is noteworthy that other markers of senescence, such as the activation of the DDR, the upregulation of p21 or p16 have not been assessed in these studies, and the definition of immunosenescent cells is based exclusively on surface marker expression. To understand their physiological or pathological effects, it would be important to further characterise other features of senescent cells in these T cell populations, including secretory phenotype.

HCV is an enveloped positive single-stranded RNA virus that infects the liver, inducing acute hepatitis. However, 70–80% of infected individuals develop a chronic infection, which can result in liver fibrosis, cirrhosis, and hepatocellular carcinoma (HCC) [60]. Memory T lymphocytes isolated from the peripheral blood of HCV-positive patients present shorter telomeres compared to healthy subjects [61]. In addition, CD8+ T cells show a sign of DNA damage as assessed by the detection of γH2AX, and this subpopulation does not phosphorylate STAT1 and STAT5 in response to IL-6 or IL-2 stimulation, respectively, compared to unfractionated CD8+ T lymphocytes [62]. These data suggest that chronic HCV infection promotes T cell immunosenescence, and as discussed above, a more in-depth analysis of the senescence markers will contribute to a better characterisation of their contribution to the disease progression.

The enveloped positive single-stranded RNA retrovirus HIV targets mainly CD4+ T lymphocytes and results, if not treated, in a progressive loss of this cell population. The loss of the CD4+ T population results in severe immunodeficiency characterised by susceptibility to infections and a certain type of infection-associated cancer (Kaposi sarcoma and B cell lymphoma), a syndrome known as acquired immune deficiency syndrome (AIDS). The introduction of the anti-retroviral therapy (ART) resulted in the suppression of viral replication, and strong reduction of risk to develop AIDS [63].

Recent studies demonstrate that HIV patients present T cell abnormalities similar to those identified in elderly individuals, including the expansion of CD28 negative T cells, lower naive/memory ratios, and reduced responsiveness to vaccine [64]. The expression of p16 is elevated in non-ART-treated patients compared to uninfected subjects, and does not correlate with chronological age, suggesting that active HIV infection is associated with immunosenescence. ART treatment reduces the levels of p16 expression to those observed in healthy controls in the CD4+ compartment, but not in CD8+ T lymphocytes [65,66].

Interestingly, HIV-specific CD8+ T cells have significantly shortened telomeres in patients with progressive disease (progressors) compared to those observed in the limited group of HIV-positive individuals who do not show an increase viremia (controllers). Blockage of the immunological checkpoint with anti-PD-L1 specific antibody, promotes the upregulation of the telomerase and consequent telomeres elongation, suggesting that the checkpoint inhibitor therapy may enhance immune control against HIV-1 [67].

Similarly to HIV, the enveloped positive single-stranded RNA virus HTLV-1 infects CD4+ T lymphocytes, and it is the causative agents of two diseases: acute T-cell leukaemia/lymphoma (ATLL) and a demyelinating disease known as HTLV-1-associated myelopathy/tropical spastic paraparesis (HAM/TSP) [68]. HTLV-1-infected cells isolated form the peripheral blood of ATLL patients, and HTLV-1 transformed cell lines, show signs of telomere attrition. In the latter case, telomere shortening is associated with the presence of telomere dysfunction-induced foci (TIF) and ATM phosphorylation: both are characteristic signs of RS [69,70].

### 3.2. Effect of Senescence in Microbial Replication and Invasion

The number of persons over 60 years of age is expected to double from 11% of the world’s population to 22% by 2050 [71]. An extended lifespan is also coupled with an increased susceptibility to infection which can be associated with the declined efficacy of the immune system, as discussed in the previous section. However, recent studies indicate that pathogenic bacteria exploit senescent cells to establish infections in ageing organisms, thus the increased susceptibility of the elderly to infection may be multifaceted.

#### 3.2.1. Bacterial Infections

In rats, the aged animals are more susceptible to infection by *Salmonella enterica* serovar Typhimurium [72], which invades the cells of the intestinal mucosa and is a leading cause of human gastroenteritis [73]. The increased susceptibility may be due to senescence-induced increased caveolin-1 expression observed in ageing humans, rats and mice, which promotes enhanced *Salmonella* uptake in non-phagocytic senescent fibroblasts in vitro [74].

RS and caveolin-1 have also been demonstrated to enhance the invasion of *Fusobacterium nucleatum* in primary human gingival fibroblasts (GFs) [75]. Interestingly, in these settings *F. nucleatum* infection promotes a reduction in IL-6 and IL-8 secretion in senescent cells compared to infected low passage GFs, adding additional evidence to the fact that SASP can be differentially regulated under certain conditions, such as infections.

Lower respiratory tract infection by *Streptococcus pneumoniae* is a leading cause of infection-related fatalities in the elderly [76]. Age-associated inflammation in the lung is linked with increased senescence markers (e.g., p16 expression) and SASP (e.g., IL-1α/β, IL-6, TNF-α and CXCL1), which is coincident with an increased expression of *S. pneumoniae* cell-surface receptors keratin 10 (K10), laminin receptor (LR) and platelet-activating factor receptor (PAFr) [77]. Senescent cells with increased K10 and LR are more permissive for infection *in vitro*. In accordance with the in vitro data, the induction of genotoxin stress in mice via the administration of bleomycin or H_2_O_2_ enhances the levels of p16, LR, and pro-inflammatory cytokines in the lungs, as well as the susceptibility to pneumococcal infection [77]. The upregulation of senescence markers in old mice (p21) and the downregulation of *S. pneumoniae* receptors (LR, K10 and PAFr) could be reversed by enteric administration of rapamycin, a known inhibitor of the mTOR pathway, resulting in decreased tissue damage in the lungs and the increased survival of mice upon infection [78].

#### 3.2.2. Viral Infections

The elderly population is very susceptible to infection by the negative single-stranded RNA influenza virus (IV) infection and the reactivation of the double stranded DNA varicella zoster virus (VZV) [79,80]. Besides the reduced efficacy of the immune response, the increased susceptibility of viral infection could be fuelled by an enhanced viral replication in senescent cells.

Recent evidence showed that primary human bronchial epithelial cells or human dermal fibroblasts undergoing RS present increased susceptibility to both IV and VZV as assessed by increased viral gene expression and virus titres compared to non-senescent cells [81]. The higher susceptibility of senescent cells to viral infection could possibly be exploited to eliminate chemotherapy-induced senescent cancer cells, which in vitro have been shown to undergo accelerated lysis upon infection with the measle vaccine virus (MeV, a virus-dependent senolysis), thus suppressing the potential pro-tumorigenic effects of the chronic SASP in the tumour microenvironment [82]. It is noteworthy that infection with the MeV was performed on a panel of cancer cell lines 72 h after treatment with chemotherapeutic drugs, such as doxorubicin, taxol and gemcitabine, and although senescence was confirmed by SA-β-gal staining, it would be interesting to assess the virolytic effects after a longer period of time.

The enhanced susceptibility of senescent cells to viral infections may be a direct consequence of their ability to resist apoptosis and tolerate a high ER protein load, and/or it could be dependent on an increased expression of the virus-specific receptors on the host cell surface. Interestingly, ACE-2, the receptor for the SARS-CoV-2 virus [83], and the angiotensin system promote senescence in coronary artery endothelial cells [84]. Whether this effect is associated with an enhanced viral entry and replication in senescent cells has not been experimentally assessed, but it is an important aspect to be addressed and may explain the high morbidity and mortality in elderly individuals.

Cellular senescence can also represent an innate defence mechanism to limit microbial replication. This has been demonstrated for the negative-sense single-stranded RNA vesicular stomatitis virus (VSV), which presents a significantly reduced ability to replicate in senescent mouse embryonic fibroblasts (MEFs) or in tumour cell lines, such as A549 or MCF7. The biological relevance of this finding has been validated in an in vivo model, where the induction of genotoxicity and consequent senescence by the administration of bleomycin reduces viral recovery from the lungs of VSV-infected mice 6 days post-infection [85]. Similarly, the infection of human primary dermal fibroblasts with the double stranded DNA merkel cell polyoma virus (MCPyV) activates the ATM-dependent DDR response and KAP1-dependent senescence, resulting in a significant reduction of the viral replication rate [86].

### 3.3. Microorganism-Induced Interference of Senescence

#### 3.3.1. Induction of Senescence

Given the evidence that pathogenic bacteria exploit senescence to establish infections in ageing organisms, it seems possible that bacteria have evolved virulence strategies to induce premature senescence in target cells. Strategies may include inducing senescence indirectly via persistent inflammatory responses [23]. For example, pathogen-associated molecular pattern (PAMP) molecules, such as bacterial lipopolysaccharide, have been shown to induce stem cell senescence by the persistent activation of the pattern recognition receptor Toll-like receptor 4 through the NFκB-p53-p21 signalling axis [87]. While it remains unclear whether bacteria exploit PAMPs as an indirect means to hijack inflammation-mediated senescence, dedicated microbial genotoxins are becoming increasingly implicated in direct induction of senescence. Bacterial genotoxins are effectors that cause DNA breaks in mammalian cells [23,88,89]. Intoxication results in DDRs that orchestrate repair and cell fate decisions including survival. However, if the DNA damage is too extensive, the damaged cells die by apoptosis or enter senescence.

Colibactin produced by the B2 lineages of the enteric pathogen *Escherichia coli* causes DNA replication stress during mammalian cell infection by inducing DNA interstrand cross-links, which recruit the DNA cross-link repair Fanconi anemia protein D2 (FANCD2) and activates the ATM-and-Rad3 related (ATR) kinase [90,91]. Infected cells often undergo cell death, but a significant proportion display hallmarks of cellular senescence including enhanced SA-β-gal activity, the expansion of PML bodies and senescence-associated heterochromatic foci (SAHF) [22]. The idea that colibactin induces senescence was reinforced by evidence of SASP characterised by the secretion of proinflammatory molecules (i.e., IL-6, IL-8, MCP1, MMP3) and transmissible senescence in bystander cells that promoted cancer cell proliferation [22].

Foremost amongst bacterial genotoxins is the family of cytolethal distending toxins (CDTs) encoded by diverse Gram-negative pathogenic bacteria. CDTs comprise a genotoxic CdtB subunit with functional and structural homology to mammalian DNase-I, which is non-covalently linked to the accessory CdtA and CdtC subunits that mediate CDT internalisation [92,93,94]. CDT nuclease activity is thought to induce double-strand breaks (DSBs) and single-strand breaks (SSBs) in DNA that trigger DDR, including ATM/ATR activation, cell cycle arrest and cellular distension [95,96,97,98,99]. Persistent γH2AX/53BP1 DDR induced by *Haemophilus ducreyi* CDT was shown to induce the hallmarks of premature cellular senescence in normal and cancer cell types [17]. The hallmarks included enhanced SA-β-gal activity, the expansion of PML nuclear bodies and the expression of cytokines IL-6, IL-8, and IL-24. The authors likened the CDT-induced senescence to an anti-cancer response that highlights the potentially oncogenic effects of bacterial genotoxins. Consistent with this, the heterologous expression of CdtB triggered a senescence-like response in tumour xenografts in mice [100].

While the direct induction of senescence by genotoxins further implicates bacteria in oncogenesis, it is less clear whether senescence is subverted by pathogens to promote infection. One example recently implicated in senescence includes the typhoid toxin [19], which is a unique chrimeric toxin encoded by the serovars of the intracellular pathogen *Salmonella* that cause enteric fever (e.g., typhoidal serovars *S.* Typhi, *S.* Paratyphi) and inflammatory gastroenteritis (e.g., non-typhoidal serovars *S.* Javiana, *S.* Montevideo) in humans [101,102,103]. The typhoid toxin has been reported to play important roles during the *Salmonella* infection of mice and humans including pathogen dissemination and host colonisation, the suppression of inflammatory responses, bacteraemia, typhoid fever and chronic *Salmonella* carriage [103,104,105,106]. The typhoid toxin mediates its toxigenic activities through CdtB, which is exocytosed from infected mammalian cells in a chimeric complex with the pertussis toxin subunits PltA and Plt, the latter facilitate toxin endocytosis into bystander cells where CdtB causes DNA damage [102,103,107]. Recent work on the typhoid toxin revealed that senescence is hijacked by *S.* Typhi and *S.* Javiana to enhance the intracellular *Salmonella* infection of macrophage and fibroblast-like cells [19]. A senescent-like phenotype was triggered by two mutually exclusive DDRs in distinct cell populations: ATM-dependent γH2AX foci in G1 or ATR-dependent γH2AX localised at the nuclear periphery in G2/M, referred to as RING (response induced by a bacterial genotoxin). The RING phenotype was a result of excessive single-strand DNA breaks that sequestered the replisome component replication protein A (RPA) and caused DNA replication stress. Consistent with this, RPA exhaustion by small molecule inhibitors is known to cause replication catastrophe leading to cellular senescence [108]. The toxin-induced DDRs led to SASP-containing unidentified factors, which caused transmissible senescence in bystander cells that were more susceptible to *Salmonella* infection [19]. This suggests that pathogenic bacteria hijack senescence to modulate the infection niche as part of their virulent strategy, as discussed below.

#### 3.3.2. Inhibition of Senescence

While certain bacteria may actively promote senescence, other microorganisms have been shown to negatively interfere with this process either to promote the proliferation of the host cells to ensure their replication or to expand the pool of infected cells. Interestingly, all these microorganisms are either well characterised oncogenic viruses or bacteria associated with cancer development.

A classic example is human papilloma virus (HPV), the major risk factor for cervical cancer [109]. HPV is a non-enveloped double stranded DNA virus that infects cutaneous and mucosal epithelia and relies on the host DNA replication machinery to ensure the replication of its genome. The initial infection occurs within the dividing cells of the skin basal layers or the ectocervix. When the infected cells differentiate and stop proliferating, the expression of the viral gene product E7 activates cell cycle progression, by binding to and promoting the degradation of the tumour suppressor protein RB. However, the unrestrained proliferation imposed by E7 induces OIS in the infected cells. This event is prevented by the HPV E6 protein, which promotes p53 degradation. Based on their effects on the cell cycle progression, E6 and E7 are classified as viral oncogenes [109].

Epstein–Barr virus (EBV) and Kaposi sarcoma virus (KSV) are oncogenic viruses belonging to the family of double stranded DNA gamma herpes viruses. Both establish a latent infection in B cells (EBV and KSV) or in the blood and lymphatic endothelial cells (KSV), characterised by the expression of a limited number of viral proteins [110,111]. Initially, latent infection is believed to promote a strong proliferative burst of the infected cells to enhance the virus pool, which can further reside in the infected cells in a non-productive form (characterised by the expression of a limited number of viral genes, known as latent antigens), until the switch to a lytic type of infection, where new virions are produced.

The proliferative burst induced by EBV and KSV has to overcome the RS, and these viruses have adopted different strategies to negatively regulate this process. The initial infection of primary B cells with EBV is associated with telomere erosion and the activation of telomeric DNA damage. However, a proportion of these cells will activate a telomerase-independent mechanism, known as the alternative lengthening of telomeres (ALT), regulated by the latent protein EBNA-1, and followed by the activation of human telomerase (hTERT) at a later stage in EBV-immortalised lymphoblastoid B cells (LCLs) [112,113]. A recent paper has demonstrated that the KSV promotes the proliferation of lymphatic endothelial cells beyond the replicative senescent limit, and this process is driven by the expression of the viral protein vCyclin, a homologue of the cellular cyclins D and E [20].

The transforming capacity of these viruses is a collateral damage of the pro-proliferative feature associated with anti-apoptotic and anti-senescence responses in combination with the failure of the host safeguard mechanisms due to: (i) the integration of the viral HPV genome and the constitutive expression of the viral E6 and E7; (ii) the acquisition of additional mutations that escalate the process of immortalisation to transformation (EBV, KSV); (iii) the lack of immune control of EBV-transformed LCL as consequence of immunosuppression.

Acute infection with *Chlamydia trachomatis* in E1A immortalised fibroblasts has been associated with the increased expression of TERT, resulting in: (i) a slight increase in telomere length, which was sustained even after clearance of the bacterial infection; and (ii) a reduced telomeric γH2AX phosphorylation in late passage cells [114]. These effects could promote the survival of cells with a higher potential to acquire genomic instability, providing one possible molecular mechanism to support the epidemiological correlation between *C. trachomatis* infection and cervical cancer [115]. It would be interesting to assess whether the enhanced TERT expression is required to establish a suitable niche for *C. trachomatis* replication.

### 3.4. Bacterial-Induced Senescence and Modulation of the Tissue Microenvironment

A relevant biological question concerning the evolution of bacterial genotoxins and the consequent induction of senescence, is what role they exert in the context of infection and colonisation. Recent evidence has demonstrated that a functional typhoid toxin prevents intestinal inflammation in a murine model of *Salmonella enterica* [104], in spite of the induction of epithelial and stromal senescent cells during an acute infection (Martin and Frisan, unpublished data). This effect is associated with the activation of a tissue repair-type of immune response, characterised by the presence of alternatively activated-like macrophages and Th2 cytokines, such as IL-9, IL-10, and IL-13 (Martin and Frisan, unpublished data). Infection with the genotoxigenic *Salmonella* favours the establishment of a persistent infection in the liver, however, whether this effect is associated with the induction of senescence has not yet been addressed [104]. These data suggest that during the course of a natural infection, the effect of the genotoxin-induced senescence remodels the host response to promote a favourable niche for the successful stealth invasion and establishment of chronic asymptomatic carriers as a pathogen reservoir.

*Clostridium difficile* is the causative agent of antibiotic-associated diarrhoea and pseudomembranous colitis. The key virulence factors in the pathogenesis of *C. difficile* are two protein toxins, the *C. difficile* toxins A and B (TcdA and TcdB), which glucosylate and inactivate the small GTPases of the RHO family (RHOA, RAC and CDC42), promoting cell rounding, loss of adhesion and apoptosis [116]. A novel interesting aspect of TcdB is the induction of a senescence phenotype in enteric glial cells (EGC) that survive the acute cytotoxicity [117]. The phenotype, which is AKT and JNK-dependent, may contribute to the development of irritable bowel syndrome (IBS) and inflammatory bowel disease (IBD) as a consequence of *C. difficile* infection, via the prolonged secretion of pro-inflammatory mediators [117].

A senescence-dependent remodelling of the microenvironment been demonstrated also for colibactin in the context of carcinogenesis. The exposure of cancer cells to colibactin producing *E. coli* enhances tumour growth in a xenograft mouse model. This effect is dependent on the colibactin-induced senescent phenotype, following the accumulation of the sumoylated form of the tumour suppressor protein p53, and directly mediated by the microRNA miR20a-5p-induced downregulation of deSumoylase Sentrin specific protease (SENP)-1. Tumour growth is fuelled by the SASP, and specifically by the secretion of the hepatocyte growth factor (HGF). The activation of the HGF pathway and the high expression of miR20a-5p has been further observed in an azoxymethane/dextran sodium sulfate (AOM/DSS) mouse model of colorectal carcinoma (CRC) and in CRC human biopsies positive for *E. coli* producing colibactin [18].

## 4. Concluding Remarks

The current knowledge on the crosstalk between cellular senescence and infection is still in its infancy, but it highlights that senescence can exert a dual role in infection, either enhancing susceptibility to infection by: (i) promoting immunosenescence; (ii) favouring microbial replication within the host cells; (iii) the remodelling of the tissue microenvironment, or acting as part of the innate immune defence thus restricting pathogen replication. The yin and yang effect may be dependent on multiple variables, including the origin and differentiation stage of the infected cells, the microorganism, the tissue microenvironment and type of infection (acute vs. persistent).

Many questions still need to be addressed, including a multi-markers characterisation of T cell immunosenescence in chronic infections, the detection of senescence and its role in in vivo infections, the detailed analysis of the SASP and whether different conditions/infections would promote different types of secretory profiles. The technological development with the possibility to perform multiplex analysis ex vivo and in situ (single cell sequencing, mass cytometry, multiplex transcriptomics) as well as the use of senolytic compounds will allow researchers to address these issues.

Identifying the role of senescence in infections may provide the rational to design therapeutic interventions based on senolytics to eliminate senescent cells as a mean to manage persistent infections or overwhelming host immunoresponses as recently suggested for HIV and SARS-CoV-2 [118,119,120].
